# Polyploid giant cancer cells: origin, possible pathways of formation, characteristics, and mechanisms of regulation

**DOI:** 10.3389/fcell.2024.1410637

**Published:** 2024-07-11

**Authors:** Pan Liu, Lili Wang, Huiying Yu

**Affiliations:** ^1^ Laboratory of Basic Medicine, General Hospital of Northern Theater Command, Shenyang, Liaoning, China; ^2^ Beifang Hospital of China Medical University, Shenyang, Liaoning, China

**Keywords:** polyploid giant cancer cells, giant cell cycle, epithelial-mesenchymal transition, apoptosis, autophagy, senescence

## Abstract

Polyploid giant cancer cells (PGCCs) are characterized by the presence of either a single enlarged nucleus or multiple nuclei and are closely associated with tumor progression and treatment resistance. These cells contribute significantly to cellular heterogeneity and can arise from various stressors, including radiation, chemotherapy, hypoxia, and environmental factors. The formation of PGCCs can occur through mechanisms such as endoreplication, cell fusion, cytokinesis failure, mitotic slippage, or cell cannibalism. Notably, PGCCs exhibit traits similar to cancer stem cells (CSCs) and generate highly invasive progeny through asymmetric division. The presence of PGCCs and their progeny is pivotal in conferring resistance to chemotherapy and radiation, as well as facilitating tumor recurrence and metastasis. This review provides a comprehensive analysis of the origins, potential formation mechanisms, stressors, unique characteristics, and regulatory pathways of PGCCs, alongside therapeutic strategies targeting these cells. The objective is to enhance the understanding of PGCC initiation and progression, offering novel insights into tumor biology.

## 1 Introduction

Tumors, arising from the aberrant proliferation and differentiation of normal cells, exhibit diverse genomic compositions and molecular phenotypes, contributing to significant heterogeneity within the tumor cell population (Herbein and Nehme, 2020). Chromosome instability (CIN) is a critical factor in tumor heterogeneity during progression, fostering treatment resistance, recurrence, and metastasis (Bakhoum and Landau, 2017). Polyploid cells, characterized by multiple sets of chromosomes, are essential for normal growth, differentiation, and response to adverse conditions and damage (Pandit et al., 2013). In physiological conditions, these cells can enhance tissue metabolic capacity and contribute to energy accumulation (Lee et al., 2009). Polyploid cells are present in various human tissues, including trophoblast giant cells (TGC), blood megakaryocytes, hepatocytes, skeletal muscle cells, and cardiomyocytes (Øvrebø and Edgar, 2018). Polyploidy has been shown to either impede or promote disease progression, tissue repair, and regeneration (Vainchenker and Raslova, 2020). Recent studies have demonstrated a significant increase in polyploid cell numbers upon exposure to stressors such as radiation, chemicals, or other cytotoxic agents, leading to drug resistance (Zhang et al., 2014a; Zhang Z. et al., 2021; You et al., 2022). In 2018, Bielski et al. identified nearly 30% polyploid genomes in 9,692 prospectively sequenced terminal cancer patients (Bielski et al., 2018). In 2019, Priestley et al. discovered that 56% of metastatic solid tumor genomes exhibited polyploidy in a pan-cancer study (Priestley et al., 2019).Polyploid Giant Cancer Cells (PGCCs), a hallmark of chromosomal instability and diversity within tumors, may contribute to the complexity of tumor genetic makeup and development. The critical role of PGCCs in genomic instability, tumorigenesis, metastasis, drug resistance, and post-treatment tumor regeneration is increasingly recognized (Herbein and Nehme, 2020).

However, research on PGCCs remains relatively limited. This review examines the potential formation pathways, distinct characteristics, and regulatory mechanisms of PGCCs, with the aim of enhancing the understanding of their genesis and development.

## 2 Polyploid giant cells in tumor

PGCCs, also known as multinucleated giant cells (MNGCs), are referred to by various terms, including multinucleated cancer cells, hyperdiploid cells, embryonic-like cancer cells, osteoclast-like cancer cells, pleomorphic cancer cells, large cancer stem cells (CSCs), and poly-aneuploid cancer cells (PACCs) (Pienta et al., 2022). Unlike polyploid cells in developmental and inflammatory states, PGCCs possess the capacity for stemness, dedifferentiation, and regeneration. The presence of PGCCs in tumor tissues is indicative of advanced tumor grade and poor prognosis (Fei et al., 2019a).

### 2.1 Origin and identification of PGCCs

More than 180 years ago, tumor cell biologists observed and documented the presence of PGCCs in cell cultures (Amend et al., 2019). PGCCs represent a small cluster of specialized cells within the tumor cell population. These cells possess a large cytoplasm and significantly increased volume. They typically exhibit pronounced nuclear atypia, a high nuclear-cytoplasmic ratio, dark nuclear staining, prominent nucleoli, and either a single giant nucleus or multiple nuclei. The shape of their nuclei is irregular, and the volume of the nucleus is at least three times greater than that of a normal diploid tumor cell nucleus (Zhang et al., 2013a). PGCCs have been demonstrated to possess tumor stem cell properties, including the ability to form spheroids from single cells, thereby promoting heterogeneity in solid tumors (Zhang et al., 2014b). Additionally, PGCCs can produce tumors in mice following inoculation (Tagal and Roth, 2021), exhibit high plasticity, and transform into various phenotypes (Fei et al., 2019b). These cells express typical markers of both normal stem cells and CSCs, such as CD44 and CD133, and can differentiate into various tissues, including adipose tissue, cartilage, bone tissue, erythrocytes, and fibroblasts under specific culture conditions (Zhang et al., 2014a). Recent research suggests that PGCCs are not degenerative or conventional CSCs but rather represent a transitional phase between stem cells and fully developed tumor cells, potentially resembling blastomere-like CSCs (Salem et al., 2020). Furthermore, PGCCs are a hallmark of oncovirus infection and are critical in the process of virally induced tumors (Herbein and Nehme, 2020).

PGCCs were initially thought to arise from repeated failures of mitosis or cytokinesis. Recent research suggests that PGCCs are not permanently matured aged cells incapable of cell division but rather a collection of abnormal cells capable of generating viable offspring crucial in all phases of cancer development and progression (Moein et al., 2020). While most stress-induced polyploid cells perish, a small number of surviving polyploid cells remain active and possess the capacity to proliferate, accurately distributing the chromosomes of new cancer cells through mechanisms such as asymmetric division, cleavage, fission, budding, splitting, and rupture, resulting in the creation of normal-sized diploid descendant cancer cells. This process is known as reductive depolyploidization (Liu, 2018). Consequently, PGCCs are believed to possess the capability of acting as stem cell-like cells, generating offspring via intranuclear mitosis following mitotic catastrophe (Erenpreisa et al., 2005). Offspring generated by PGCCs may exhibit mesenchymal cell characteristics and possess enhanced migratory and invasive capabilities compared to typical diploid tumor cells (Zhang et al., 2013b).

PGCCs can be detected in numerous types of cancer, including breast, ovarian, colorectal, glioblastoma, melanoma, lung, pancreatic, bladder, kidney, thyroid, and prostate cancer (Amend et al., 2019; Casotti et al., 2023). One notable characteristic of high-grade serous ovarian cancer (HGSOC) is the presence of mononucleated or multinucleated PGCCs (Liu, 2018). Cobalt chloride (CoCl_2_), a chemical hypoxia inducer that selectively kills common diploid tumor cells, has been used to induce a variety of cell lines to produce PGCCs and their progeny. Studies have shown that PGCCs induced by CoCl_2_ play a crucial role in cancer progression, including invasion, metastasis, and chemotherapy resistance (Zhang et al., 2014a). It has been demonstrated that ovarian cancer cells treated with CoCl_2_ can transform into PGCCs and gain the capacity to produce diploid progeny cells through processes such as budding and division (Zhang et al., 2014a). Furthermore, PGCCs generated after CoCl_2_ exposure were found to be morphologically larger, with multiple nuclei or a single giant nucleus, compared to normal diploid tumor cells (Fei et al., 2019c). In addition to CoCl_2_, cancer treatments can also induce cell polyploidization (Pienta et al., 2021). Chemotherapy drugs like platinum compounds, which damage DNA, and taxanes, which stabilize microtubules, can create PGCCs, while radiotherapy can also significantly enhance the occurrence of PGCCs (Alhaddad et al., 2023).

### 2.2 Possible pathways of PGCC formation

Mitosis is frequently regarded as the predominant form of cell division in mammals. It is an intricate and carefully controlled process characterized by the formation of the spindle and the alignment of chromosomes, ensuring that replicated daughter chromosomes are evenly distributed to daughter cells (Zhang et al., 2022). However, errors in chromosome segregation during this process can lead to mitotic divisions of centrosomes or spindles, potentially creating cells with abnormal numbers of chromosomes and increasing the risk of cancer development (Sotillo et al., 2007).

Cell replication and division follow a specific sequence known as the cell cycle. Cyclin-dependent kinases (CDKs) play a crucial role in controlling the cell cycle by initiating mitosis and regulating Aurora B, a checkpoint that ensures accurate chromosome segregation (Richards et al., 2021). It has been shown that varying thresholds of CDK activity can trigger the activation of the S and M phases. The S-phase threshold is typically reached by A-type or E-type cyclins complexed with CDK2, while the M-phase threshold is achieved by A-type or B-type cyclins complexed with CDK1 (Edgar et al., 2014). However, in endoreplication cell cycles, cells can become polyploid through the endocycle, where only the S-phase threshold is cyclically reached (Edgar et al., 2014). Additionally, PGCCs experience G2/M arrest, and their formation is strongly associated with the aberrant expression and altered subcellular localization of cell cycle-related proteins such as cell division cycle 25 homolog C (CDC25C) (Fei et al., 2019c). Both cell cycle checkpoint kinases, such as CHK1/CHK2, and the tumor suppressor P53 are involved in G2/M-phase arrest through the modulation of CDC25C (Thanasoula et al., 2012; Lopez-Sánchez et al., 2014). Aurora A and polo-like kinase 1 (PLK1), members of the serine kinase family, modulate CDC25C activation (Gheghiani et al., 2017). Phosphorylation of CDC25C determines its subcellular localization. Consequently, factors such as CHK1, CHK2, P53, Aurora A, PLK1, and the phosphorylation state of CDC25C influence the formation of PGCCs by modulating CDC25C expression. Liu et al. demonstrated that the CHK1, CHK2-pCDC25C-Ser216-cyclin B1-CDK1, and Aurora A-PLK1-pCDC25C-Ser198-cyclin B1-CDK1 pathways play a role in the formation and development of PGCCs (Liu et al., 2020).

**FIGURE 1:**
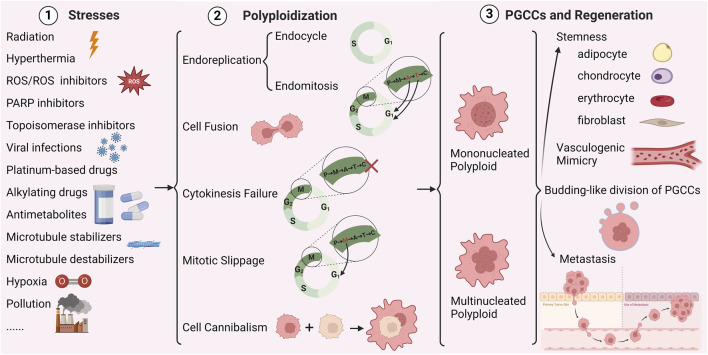
Mechanisms of polyploidy formation in cancer cells (Created with BioRender.com). P represents prophase, M represents metaphase, A represents anaphase, T represents telophase, C represents cytokinesis, × represents cytokinesis failure.

Figure 1 summarizes various studies that demonstrate how a range of stressors induce PGCCs through endoreplication, cell fusion, cytokinesis failure, mitotic slippage, and cell cannibalism (Was et al., 2022).

#### 2.2.1 Endoreplication

Endoreplication is characterized by a deviation from the typical cell cycle, wherein cells undergo multiple rounds of whole genome duplication (WGD) without subsequent mitotic division. This process results in cell enlargement, an increase in genome ploidy within the nucleus, and the formation of a large polyploid cell encompassing the entirety of the genetic material (Fox et al., 2020; Pienta et al., 2022). The endoreplication cycle of polyploids can be classified into two distinct processes: endocycle and endomitosis. Endoreplication serves as a bridge between the G2 and G1 phases, with endocycling cells transitioning between the G and S phases without undergoing mitosis or cytokinesis, ultimately giving rise to large mononuclear polyploid cells. In contrast, endomitosis involves nuclear division without accompanying cell division, leading to the formation of multinuclear polyploid cells (Liu, 2018). Recent studies have discovered that PGCCs undergo nuclear growth and division in two manners: multipolar endomitosis (MEM) and restitution multipolar endomitosis (RMEM) (Li et al., 2023). MEM is the phenomenon in which full segregation of individual nuclei occurs after genome proliferation. RMEM describes the inability of converged chromosomes to segregate after the onset of mitosis, leading to extensive nuclear fragmentation and polymorphism of the nucleus (Li et al., 2023). This indicates that instead of undergoing cytokinesis during the cell cycle, the formation of PGCCs involves multiple nuclear replication methods such as endocycle, MEM, RMEM, nuclear fusion, nuclear fragmentation, micronuclei, or nuclear motility, resulting in intracellular multiplication and a higher nuclear-cytoplasmic ratio (Li et al., 2023). DNA replication and nuclear amplification are carried out efficiently, thereby increasing the heterogeneity of PGCCs.

The process of endoreplication is evolutionarily conserved and observed in various cell types, particularly in PGCCs treated with chemotherapy. In stressful and hypoxic environments, tumor cells can undergo epigenetic and genetic alterations through endoreplication, leading to the formation of large cells with multiple genome copies to counteract cytotoxic stress and develop drug resistance (Richards et al., 2021). Endoreplication increases the DNA content in the cell nucleus, facilitating gene transcription, enhancing RNA and ribosome synthesis, increasing protein production, and generating additional energy and resources to support cell proliferation. Key mitotic regulators during endoreplication are influenced by the DREAM complex, MASTL, p-MSK1-T581, p-CTNNB1Ser-33, p-CTNNB1Ser-37, Thr41, p-mTOR-S2481, p-mTOR-S2448, and p-AMPKα-T172 through their expression and subcellular localization (Richards et al., 2021). Although the factors regulating the endoreplication process are not fully understood, changes in Aurora A and Aurora B may be involved (Niu et al., 2016). Furthermore, the Notch pathway appears to play a role in endoreplication, potentially contributing to the notable rise in CCNE1 and the decline in CCNB1 during S-phase (Lee et al., 2009). Zhang S et al. reported that endoreplication is the potential mechanism for the formation of PGCCs after CoCl_2_ treatment (Zhang et al., 2014a).

#### 2.2.2 Cell fusion

The merging of two cells or protoplasts with distinct genotypes, known as cell fusion, creates a hybrid cell. This process results in the accumulation of WGD and random mutations, which contribute to the development of aneuploid cells (Delespaul et al., 2019). During cell fusion, cells transition from having disparate nuclei to sharing identical nuclei, similar to nuclear fusion. This transition leads to the loss or random reorganization of chromosomes (Berndt et al., 2013). Common in tumorigenesis and development, cell fusion causes unstable chromosomes, DNA susceptible to damage, and aneuploid cells with uneven genetic material distribution. These effects ultimately facilitate tumor initiation, progression, drug resistance, metastasis, and malignant transformation (Delespaul et al., 2019). Fusion can occur homotypically within the same cell types or heterotypically between different cell types in a tissue (Duelli et al., 2007).

Fusion cells often exhibit increased malignant traits compared to the original tumor cells due to chromosomal abnormalities such as chromosome translocations or inversions that occur during fusion. These abnormalities lead to the development of aneuploidies, enhanced growth and spread, drug resistance, stem cell-like features, and other forms of tumor diversity (Shabo et al., 2020). The process of cell fusion is carefully controlled and can be divided into five stages: i) initiation, ii) chemotaxis, iii) adhesion, iv) merging, and v) post-fusion (Zhou and Platt, 2011). Proteins responsible for cell fusion include syncytin-1, syncytin-2, glial cell missing 1, and galectin-1, as well as additional proteins such as annexin, myosin-forming proteins, and sarcomere proteins. Cell fusion is regulated by various signaling pathways, including cAMP/PKA, MAPK, Wnt, JNK, and others (Zhang H. et al., 2021). The GCM1/syncytin-1 signaling pathway has been found to induce cell fusion during the formation of PGCCs. Furthermore, hypoxia has been demonstrated to play a role in the formation of ovarian PGCCs by activating the unfolded protein response (UPR) during cell fusion. Reducing UPR activation in ovarian cancer cells may decrease cell fusion and PGCC formation in ovarian cancer (Yart et al., 2022).

#### 2.2.3 Cytokinesis failure

The division of a cell into two daughter cells during the final stage of the cell cycle, known as cytokinesis, can be disrupted by abnormalities in cellular proteins or minor issues during mitosis. Failure of cytokinesis frequently causes elevated cell ploidy and centrosome count, which is a key factor in numerous ploidy alterations observed in various human cancers (Krajcovic and Overholtzer, 2012). Cytokinesis can be disrupted by autophagic cells, and chromatin bridges at the midbody can interfere with the signaling mechanism of Aurora B kinase, leading to cytokinesis failure (Steigemann et al., 2009). When the cytokinesis activator GTPase RhoA or its downstream pathway RhoA/ROCK is inhibited, it can reduce myosin and actin aggregation, causing dysfunction of the contractile ring and ultimately leading to cytokinesis failure (Lens and Medema, 2019). Disturbances in the regulation of the spindle assembly checkpoint (SAC) can also result in chromosome mis-segregation and failure of cytokinesis (Gandarillas et al., 2018).

#### 2.2.4 Mitotic slippage

Mitotic slippage (MS) occurs in the middle of the cell cycle when the cell exits the division cycle before the late spindle assembly checkpoint (Gentric and Desdouets, 2014). Accurate chromosome segregation requires the attachment of kinetochore bipolars to the spindle microtubule, and the SAC monitors the kinetochore-microtubule junction during this process (Musacchio, 2015). If the kinetochore bipolars fail to connect, the cell escapes from SAC-induced mitotic arrest through MS. During the transition from premitosis to metaphase, tumor cells can transition to interphase due to MS. In the process of MS, the cells enter the G1 phase without completing mitosis and cytokinesis, resulting in the formation of a giant polyploid multinucleated cell. However, these large cancer cells are unable to continuously replicate their DNA, leading to a halt in replication and the formation of polyploid senescent cells that are incapable of tumor formation (Moiseeva et al., 2015).

Paclitaxel, a type of spindle inhibitor, is often the initial choice for treating cancerous tumors. As a cell cycle inhibitor, the primary mechanism of paclitaxel involves binding to intracellular microtubule proteins, inhibiting the formation of spindle filaments by disrupting the dynamic balance of microtubule polymerization and depolymerization. This disruption causes rapidly dividing tumor cells to arrest in the G2/M phase, preventing further proliferation and ultimately initiating apoptotic programs due to growth inhibition, resulting in programmed cell death (PCD). However, if the apoptotic mechanism and/or cell cycle control is abnormal, despite the cell remaining in the mitotic phase for a short time, the cell will eventually progress to the next cell cycle, i.e., MS, and polyploidy will occur (Mantel et al., 2008). Furthermore, docetaxel (Doc), another medication that inhibits microtubules and is used to treat prostate cancer, can also cause cell cycle arrest, leading to MS. In this syndrome, tumor cells become polyploid due to chromosomal instability, resulting in the formation of giant cells with multiple nuclei and centrosomes (Ogden et al., 2015; Mittal et al., 2017).

Ruggiero et al. discovered that in budding yeast, MS is dependent on PP1 phosphatase, which facilitates MS by dephosphorylating its subunit Mad3 and destabilizing the mitotic checkpoint complex (MCC) (Ruggiero et al., 2020). Studies have shown that the inhibition of TORC1 kinase can lead to MS in both budding yeast and human cells, ultimately resulting in chromosomal instability. During MS in yeast, several changes occur, including the activation of phosphatase Cdc14, the disintegration of the nucleolar protein Net1, and the degradation of the APC/C-Cdh1-dependent enzyme inhibitor protein and cyclin B during the mid-stage (Yamada et al., 2022).

#### 2.2.5 Cell cannibalism

Cell cannibalism, also known as entosis, is a lysosomal self-digestion process where internalized cells disrupt cytoplasmic divisions in their phagocytic hosts, potentially leading to polyploidy. Documented in mammalian systems for over a century (Overholtzer and Brugge, 2008), this behavior involves one cell engulfing another, resulting in the death of the internalized cell (Fernandez-Flores, 2012). This process can contribute to tumor progression by altering chromosome numbers (Krajcovic and Overholtzer, 2012). Additionally, cannibalistic cancer cells exhibit a higher propensity for metastasis (Overholtzer and Brugge, 2008; Sharma and Dey, 2011). Thus, this act of cannibalism can be seen as a mechanism that provides cancer cells with essential nutrients (Lugini et al., 2006).

Multiple factors may contribute to CC, including mitosis (Durgan et al., 2017), glucose starvation (Hamann et al., 2017), matrix deadhesion (Overholtzer et al., 2007), hypoxia, and acidic pH (Alfarouk et al., 2011). Some researchers regard internalized cells as invaders (Overholtzer and Brugge, 2008). The structure of cannibalistic cells in human breast cancers indicates that the engulfment mechanism involving β-catenin relies on cell-cell adhesion (Overholtzer et al., 2007). During the CC process, internalized cells may act as physical obstructions in the host cytoplasm, impairing the normal completion of cytokinesis (Krajcovic et al., 2011). Consequently, the external cells may undergo polyploidization and survive, driving cancer progression (Krajcovic et al., 2011).

### 2.3 Stresses for PGCCs

Several stresses induce tumor cells to form polyploids, including genotoxic stresses such as radiation and chemotherapeutic agents commonly used in clinical oncology treatment, targeted therapies, viral infections, pulsed thermotherapy, microgravity environments, and other stimuli. These pressures can lead to DNA double-strand breaks or alterations in the levels of proteins linked to cell division and cell merging, ultimately causing the creation of PGCCs (Mosieniak and Sikora, 2010). More research is still needed to explore new factors that may induce polyploidy. For instance, some researchers have suggested that in the context of modern society’s lifestyle, factors involved in inducing the formation of PGCCs are also related to the patient’s intrinsic epigenetic factors and extrinsic exposure phenomena such as cigarette smoke, hyperthermia, ultraviolet rays, and pollution (Was et al., 2022). Table 1 shows that a variety of stresses and cellular processes are associated with the formation of polyploids in various cancer cells.

**TABLE 1:** Various types of stresses and cellular processes implicated in the development of polyploidy in different cancer cell types.

Stresses	Cancer types and cell types	Polyploidy mechanism	References
Radiation	Human cervical cancer cell line (Hela)	Endoreplication	Erenpreisa et al. (2008), Erenpreisa et al. (2009)
Alkylating agent (Mitomycin C)	Human non-small cell lung cancer cell line (A549)	Endoreplication	McKenna et al. (2012)
Platinum-Based drug (Cisplatin)	Human breast cancer cell line (MX-1)	Endoreplication	Wolf et al. (1996)
	Rat colon cancer PROb cell line (DHD-K12-TRb)	Endoreplication	Puig et al. (2008)
Antimetabolite (5-Fluorouracil)	Human triple-negative breast cancer cell line (MDA-MB-231)	Endoreplication and mitotic slippage	Milczarek et al. (2018)
Topoisomerase inhibitor (Doxorubicin)	Human colon cancer cell line (HCT116)	Endoreplication	Sliwinska et al. (2009), Mosieniak et al. (2015)
PARP inhibitor (Olaparib)	MYCN-dependent human neuroblastoma cell lines (ACN, SK-N-AS, LAN-1, and IMR32)	Endoreplication	Colicchia et al. (2017)
	Human ovarian cancer cell lines (HEY, SKOV3, OVCA-432, OVCAR8, OVCAR5, and PEO-1)	Endoreplication	Zhang et al. (2023)
	Human breast cancer cell line (MCF-7)	Endoreplication	Zhang et al. (2023)
Hypoxia (cobalt chloride)	Human colorectal cancer cell lines (LoVo and HCT116)	Endoreplication	Fei et al. (2019a), Zhao et al. (2021)
	Human colon cancer cell lines (HCT116 and Caco-2)	Endoreplication	Lopez-Sánchez et al. (2014)
	Human ovarian cancer cell lines (HEY and SKOV3)	Endoreplication	Zhang et al. (2013b)
	Human breast cancer cell lines (MDA-MB-231, MCF-7, and BT-549)	Endoreplication	Zhang et al. (2013a), Fei et al. (2015), Parekh et al. (2018)
High-risk human cytomegalovirus infection	Human breast cancer cell lines (MDA-MB-231, and MCF7)	Endoreplication	Nehme et al. (2022)
Microtubule-stabilizing agent (Docetaxel)	Human prostate cancer cell line (PC-3)	Mitotic slippage	Mittal et al. (2017)
Microtubule destabilizer (Vincristin)	Human colon cancer cell line (HCT116)	Endoreplication	Wertz et al. (2011)
Hyperthermia	Human colorectal carcinoma cell lines (HCT116, HCT15, and DLD1)	Endoreplication and mitotic slippage	Tan et al. (2019)
ROS	Human breast cancer cell lines (MCF7, MDA-MB-231, and MDA-MB-468)	Endoreplication	Roh et al. (2012), Parekh et al. (2018)
ROS inhibitor (Trolox)	Human colon cancer cell line (HCT116)	Endoreplication	Mosieniak et al. (2015)
Ultraviolet rays	Human cutaneous squamous cell carcinomas cell lines (SCC-12B.2 and SCC-13)	Endoreplication	Lee et al. (2014)

Radiotherapy involves the targeted destruction of tumor tissue by delivering high-energy radiation to tumor cells (Baskar et al., 2014). Radiation directly impacts the structure of the DNA double helix, leading to various outcomes such as cell death, senescence, and disruption of cell division in cancer cells. It also affects subcellular structures within tumor cells, including the endoplasmic reticulum, lysosomes, ribosomes, mitochondria, and other organelles, as well as the cytoplasmic membrane, tumor cell phenotype and behavior, and the immune response to tumor cells (Wang et al., 2018). Research has demonstrated that ionizing radiation can induce the formation of PGCCs and an increase in Aurora B kinase levels (Mirzayans et al., 2017).

DNA alkylation has been shown to lead to mitotic mutations, irreversible cellular senescence, and the formation of polyploids. DNA alkylating agents such as mitomycin C (MMC) play a crucial role in cancer therapy but can also induce polyploidization in cancer cells (McKenna et al., 2012). Platinum-containing medications like cisplatin can cause polyploidization in breast cancer cells, a phenomenon also observed in colon cancer cells (Puig et al., 2008). Treating triple-negative breast cancer with antimetabolites such as 5-fluorouracil (5-FU) and sulforaphane leads to cellular multinucleation and the formation of PGCCs (Milczarek et al., 2018; Was et al., 2022). Topoisomerase inhibitors like doxorubicin (DOXO) act on colon cancer and can generate polyploid cells through endoreplication (Sliwinska et al., 2009). PARP is essential for preserving centrosome stability, and inhibiting PARP can enhance centrosome amplification and promote polyploidization (Kanai et al., 2003).

Poor tumor prognosis is associated with hypoxia in tumor tissues, primarily controlled by the hypoxia-inducible factor (HIF). HIF-1α plays a crucial role in the hypoxia-responsive mechanism, leading to polyploidization in human melanoma cells. Recent studies on the role of HIF-1α in the formation of PGCCs have shown that the subcellular localization of HIF-1α can be modulated by SUMOylation at lysine residues K391 and K477. Additionally, the nuclear expression of HIF-1α can enhance the malignant phenotype of PGCCs' daughter cells (Zheng et al., 2024).

## 3 Characterizations and possible regulatory mechanisms of PGCCs

### 3.1 PGCCs and the giant cell cycle

PGCCs are a source of mitotically active cells and display unique life cycle characteristics, indicating their significant role in chemotherapy resistance and tumor development. They multiply through an unusual cell cycle known as the “giant cell cycle” or “giant cell life cycle” (Niu et al., 2016). This process consists of four distinct yet interconnected phases: the initiation phase, the self-renewal phase, the termination phase, and the stability phase (Niu et al., 2016). In the initiation phase, many G1 and G2 diploid cells die, while the surviving cells undergo nuclear replication, forming a polyploid growth pattern. The self-renewal phase follows, during which mononuclear or multinuclear polyploid giant cells emerge. In the termination phase, the nuclei of polyploid giant cells depolarize to produce diploid cells, and the giant nuclei divide into smaller nuclear vesicles or fragments. The final stage, the stability phase, sees diploid offspring cells acquiring new genetic material with varying chromosome structures, enabling them to undergo mitosis. These new tumor cells continue to divide and proliferate, increasing tumor size and resulting in a predominance of near-diploid tumor cells.

The reversible polyploidization process promotes genomic instability, a hallmark of cancer (Hanahan and Weinberg, 2011). The giant cell cycle of PGCCs facilitates the development of aneuploidy that accompanies this process (Erenpreisa et al., 2022). This large cell cycle is a crucial cellular mechanism for initiating genetic recombination, producing new tumor-initiating cells under the stress of chemotherapy, potentially leading to disease recurrence (Niu et al., 2016). The variation in chromosome structure resulting from genetic recombination serves as the foundation for natural selection during tumor progression, particularly in the acquisition of drug resistance (Heng and Heng, 2022). Therefore, the discovery of the giant cell cycle is essential for elucidating the mechanisms of progeny production, tumor recurrence, and chemotherapy resistance.

### 3.2 PGCCs and EMT

PGCCs are essential for the growth and spread of various cancerous tumors, as demonstrated by the ability of a single PGCC to initiate diverse tumor formation in living organisms (Zhang et al., 2014a). Compared to diploid tumor cells, PGCCs and their budding progeny cells exhibit reduced cytokeratin and elevated vimentin levels, indicating an epithelial-mesenchymal transition (EMT) that enhances tumor invasiveness. EMT is a critical biological process involving dynamic changes in the cytoskeleton and extracellular matrix (Bao et al., 2012). During EMT, cells produce more matrix metalloproteinases (MMPs) and convert actin microfilaments in the cytoskeleton to vimentin (Alameddine et al., 2014). Additionally, epithelial cell markers (E-cadherin) are lost, while mesenchymal cell markers (N-cadherin, vimentin, Snail/Slug, and Twist) are upregulated. Consequently, EMT leads to the loss of polarity in epithelial cells and their transition to a mesenchymal phenotype, resulting in increased migration and invasion abilities, resistance to cell death, and the ability to degrade the extracellular matrix. These changes are crucial for the progression, growth, invasion, and metastasis of tumors (Kumar et al., 2015). Furthermore, EMT can serve as a biological model to illustrate the mechanisms by which tumor cells adapt to hypoxia (Brahimi-Horn et al., 2007). In summary, the quantity of PGCCs and the presence of EMT-associated proteins strongly correlate with tumor spread and advancement.

Recent research indicates that the VEGF-CDC42-P38 MAPK signaling pathway plays a role in controlling the movement and penetration of PGCCs and their offspring (Beckers et al., 2010). VEGF acts as a tyrosine kinase receptor that mediates the activation of CDC42 (Beckers et al., 2010). CDC42, a member of the Rho GTPase group, is crucial in cancer development as it regulates cell movement, microtubule dynamics, EMT, and cell cycle progression (Tzima, 2006). Upon activation, CDC42 engages the p38 MAPK pathway, which influences the activity of other proteins and promotes cell growth, polarity, attachment, movement, and structural changes. The MAPK pathway frequently transmits signals related to cell growth, stress response, inflammation, differentiation, cancer development, metastasis, and cell death. P38 MAPK is essential in regulating cytokine signaling and stress responses (Johnson et al., 2016). Analyzing the VEGF-CDC42-p38 MAPK signaling pathway enhances our understanding of how PGCCs and their offspring invade and spread, identifies potential targets to halt cancer metastasis and relapse, and potentially extends patient lifespan. While this signaling pathway provides insight into the invasion and metastasis of PGCCs and their offspring, further investigation into its specific regulatory mechanisms is needed. Additionally, various molecular pathways contribute to the EMT process, including the Hedgehog, Notch, NF-κB, PI3K/Akt/mTOR, and Wnt/β-catenin pathways (Bao et al., 2012). Therefore, more research is required to explore the molecular processes responsible for the development of PGCCs and the EMT-related mechanisms facilitating the generation of daughter cells from PGCCs via budding.

### 3.3 PGCCs and apoptosis

Apoptosis is a form of programmed cell death that occurs in living organisms. It is regulated by both pro-death and anti-death factors, with caspase activation being a central biological aspect (Xi et al., 2022). Apoptosis is a significant response to typical genotoxic stresses, preventing cells from improperly acquiring additional copies of their genome. Conversely, malfunctions in the apoptotic process result in cells with extra sets of chromosomes, leading to inaccurate chromosome distribution, which is a major contributor to genetic instability and cancer progression. However, during normal development, some cells actively inhibit apoptosis and develop into polyploids (Herriage et al., 2024). Thus, while apoptosis may prevent polyploidy, polyploidy can actually inhibit apoptosis. Investigating the relationship between polyploidy and apoptosis in cancer could lead to improved cancer treatments (Herriage et al., 2024).

Cancer cells can evade apoptosis to multiply, and mutations in apoptosis-related genes have been discovered in numerous cancer patients, making the induction of apoptosis a key focus in cancer treatment (Marei et al., 2021; Wilson et al., 2022). However, many studies have indicated that excessive apoptotic signaling could potentially lead to cancer (Lopez and Tait, 2015; Cao and Tait, 2018; Wang et al., 2020), and increased levels of apoptosis might be linked to negative outcomes for individuals with cancer (Feng et al., 2017). Genetic material was introduced into the daughter cells of prostate cancer cells treated with Doc through the extensions of PGCCs, resulting in a significant decrease in the levels of cleaved caspase-3 and cleaved PARP apoptotic proteins in PGCCs and their daughter cells. Conversely, the levels of anti-apoptotic proteins Bcl-2, Bcl-xL, Survivin, and Beclin were notably increased compared to control cells, potentially contributing to the resistance of PGCCs to apoptosis and drug resistance in tumor cells (Mittal et al., 2017). However, X-ray exposure led to an increase in apoptosis in triple-negative breast cancer PGCCs, along with an upregulation of cleaved caspase-3 expression, indicating the diverse apoptotic characteristics of PGCCs (Zhang Z. et al., 2021).

PGCCs mainly arise from endoreplication following exposure to olaparib in ovarian cancer cell lines. However, the combined use of mifepristone and olaparib can effectively inhibit the endoreplication and survival of PGCCs, enhancing anti-tumor effectiveness (Zhang et al., 2023). Consequently, PGCCs represent promising targets for PARP inhibitor-resistant ovarian cancer, and ongoing clinical trials of mifepristone combined with PARP inhibitors offer new prospects. Meanwhile, in acute megakaryoblastic leukemia (AMKL), p21-activated kinase 1 (PAK1) is significantly enriched. Inhibition of PAK1 kinase activity retards AMKL cell growth and partially promotes polyploidization of AMKL cells, ultimately inducing apoptosis (Wang et al., 2022).

### 3.4 PGCCs and autophagy

Autophagy, an evolutionarily conserved process, plays a crucial role in maintaining cellular balance, and disruptions in autophagy can contribute to tumor development (Xi et al., 2022). It has been suggested that autophagy may facilitate the survival of tumor cells after genotoxic injury (Niu et al., 2016). According to Li et al., cells must initiate autophagy during mitosis, leading to a notable increase in the LC3-II/LC3-I ratio in polyploid cells (Li et al., 2016). Additionally, the levels of the autophagy-specific substrate p62 protein decreased significantly, indicating the presence of autophagy. During mitosis, there is an increase in the expression of autophagy-related proteins, with higher levels of Beclin-1 potentially promoting active autophagic flow in the initial phase of mitosis (Li et al., 2016). Increased autophagy may contribute to asymmetric mitosis in polyploid cells. Autophagy protects cells by sequestering cytoplasmic proteins and dysfunctional organelles for essential metabolic needs. This robust protective function allows cells to react to various internal or external triggers (Feng et al., 2014). It is believed that higher levels of autophagy might be necessary to eliminate permanently damaged DNA fragments, organelles, and proteins from cells while producing offspring from PGCCs (Bowers et al., 2022). Consequently, autophagy could serve as a defense mechanism against polyploid cells in cancer treatment, aiding in the depolyploidization and growth control of polyploid cells.

A recent study on PGCCs and autophagy has highlighted the biological properties of PGCCs, including mitochondrial changes, and identified autophagy as a key mechanism for the induction of PGCCs (You et al., 2022). Experiments with mice demonstrated that blocking autophagy, either through drugs or genetic manipulation, significantly reduces the development of PGCCs. This reduction leads to a notable decrease in metastasis and improved survival rates. Chemotherapeutic drugs damage mitochondria, activating autophagy through the AMPK-mTOR pathway, which supports the creation of PGCCs (You et al., 2022). Further analysis revealed a link between certain inactive PGCCs and the increased risk of recurrence in nasopharyngeal cancer (NPC). A greater quantity of PGCCs is associated with a shorter time to recurrence and poorer survival in NPC patients (You et al., 2022). Therefore, preventing the formation of treatment-induced dormant PGCCs may reduce recurrence and metastasis in NPC patients.

Previous studies have identified PGCCs in high-grade serous carcinoma (HGSC) and demonstrated that chemotherapy can induce autophagy, which may help cells survive drug toxicity and develop resistance (He et al., 2015). Although autophagy increases during the formation of PGCCs (Bojko et al., 2020), the interaction between drugs that modulate autophagy and the adaptability of tumor cells in the form of PGCCs has not been thoroughly explored. Research on treatments affecting the development of ovarian cancer colonies from PGCCs has suggested that autophagy might not be as critical as once thought. Autophagy inhibitors do not prevent PGCC formation but do reduce the number of offspring they produce. Additionally, controlling autophagy greatly impairs the ability of PGCCs to form colonies in HGSC (Bowers et al., 2022). Studies on ovarian cancer have shown that cisplatin triggers autophagy in cancer cells, and inhibiting autophagy enhances cisplatin-induced apoptosis (Zhou et al., 2018). Research on breast cancer PGCCs indicates elevated levels of autophagy markers p62/SQSTM1 and LC3-II (Bojko et al., 2020). Recent findings suggest that modulating autophagy does not impact the formation of chemotherapy-induced PGCCs or primary PGCCs, implying that autophagy may not be essential for PGCC formation (Bowers et al., 2022). More research is needed to further understand the relationship between PGCCs and autophagy.

### 3.5 PGCCs and stemness characteristics

PGCCs are classified as tumor-initiating cells and express markers associated with CSCs such as CD24, CD44, CD133, ALDH, and Ep-CAM (McNeely et al., 2020). The significant presence of NANOG, OCT-4, and SOX-2 indicates that PGCCs possess the capacity for diverse differentiation and self-renewal similar to that of stem cells (Niu et al., 2017). PGCCs and their daughter cells have the ability to transform into various types of stromal cells, including fibroblasts, adipocytes, chondrocytes, endothelial cells, and myoepithelial cells (Zhang et al., 2014a). Therefore, targeting specific proteins or signaling pathways involved in the generation and transformation of PGCCs could offer potential avenues for treating solid tumors. Furthermore, studies have demonstrated that a single PGCC can generate spherical structures and produce xenografts, indicating the tumorigenic potential of PGCCs (Zhang et al., 2014a). PGCCs exhibit asymmetric cell division, involving budding, splitting, and bursting as growth patterns. Daughter cell budding typically occurs in the extensions of PGCCs and PGCCs with numerous nuclei (Zhang et al., 2014a). Daughter cells derived from PGCCs may develop enhanced invasion, metastasis, and resistance to chemoradiotherapy.

Furthermore, the presence of tumor budding and micropapillary structures is strongly associated with PGCCs and their daughter cells. The stemness of PGCCs is also characterized by angiogenesis. PGCCs can be observed in most microcapillary structures and are capable of generating red blood cells and creating vasculogenic mimicry (VM). These red blood cells contain fetal and embryonic hemoglobin following exposure to CoCl_2_ (Zhang et al., 2013a). The strong oxygen affinity of embryonic hemoglobin enables cancer cells to thrive in harsh hypoxic environments (Kazazian and Woodhead, 1973; Huehns and Faroqui, 1975; Peschle et al., 1985; Albitar et al., 1992).

### 3.6 PGCCs and senescence

Cellular senescence is often defined as a state in which the cell cycle is arrested and the ability to proliferate is reduced (Sapega et al., 2018). This state is also reflected in PGCCs, showing similarities between senescent cells and PGCCs. First, both are caused by DNA damage and stimulate the activation of tumor suppressors P53 and RB. Both can inhibit cell death by controlling P53 activity and/or the production of anti-apoptotic proteins. Additionally, both involve p21 or ROS factors and re-express stemness genes (Gorgoulis et al., 2019; Guillon et al., 2019; Saleh et al., 2019; Moiseeva et al., 2022). Certain PGCCs can bypass senescence-induced cell cycle arrest, generating near-diploid offspring that exhibit increased aggressiveness and resistance to genotoxic therapies via budding or asynchronous division (Puig et al., 2008; Niu et al., 2017). This enables cancer cells to evade senescence triggered by various stressors such as treatment, nutrient deprivation, or hypoxia (Zhang et al., 2013b; Coward and Harding, 2014; Zhang et al., 2022). Chemotherapy can induce cellular senescence, a distinct outcome from cell demise that may be associated with PGCCs (Sikora et al., 2022). Senescence is linked to genomic instability that leads to polyploidy (Mirzayans et al., 2010). The current belief is that the p53 pathway and the p16/pRb pathway play major roles in cellular senescence (Bharadwaj and Mandal, 2020). PGCCs display characteristics similar to senescent cells, such as a non-dividing flat mast cell shape, irreversible cell cycle arrest, increased levels of p21, Ki-67, p-histone H2A.X, and heightened activity of senescence-associated β-galactosidase (SA-β-gal) (Mirzayans et al., 2018; Zhao et al., 2023). Some PGCCs do not express SA-β-gal during senescence but instead generate CSCs through budding and rupture (Mosieniak et al., 2015). Both SA-β-gal positive and negative senescent cells can be found in PGCCs, indicating a close yet distinct relationship between polyploidy and senescence (Saleh et al., 2022).

The presence of extra chromosomes and cellular aging are essential for PGCC regeneration. Cells that age without polyploidy may not resume division (Saleh et al., 2022; Sikora et al., 2022). After senescence, PGCCs release cytokines, chemokines, and growth factors that control the tumor microenvironment, known as the senescence-associated secretory phenotype (SASP) (Bharadwaj and Mandal, 2020). SASP factors enhance tumor cell growth, invasion, movement, blood vessel formation, and EMT (Soto-Gamez and Demaria, 2017). PGCCs trigger the p44/42 pathway in neighboring tumor cells via SASP, leading to resistance (Parekh et al., 2018). Additionally, senescent PGCCs release substances like VEGF and MIF when exposed to high levels of ROS, enhancing nearby tumor cell survival (Parekh et al., 2018).

New evidence indicates that blocking IL-1β can decrease p-histone H2A.X (γ-H2A.X) levels and promote polyploidy, ultimately enhancing the pro-apoptotic effects of Doc (Zhao et al., 2023). IL-1β inhibition and Doc induction synergistically promote PGCC formation. IL-1β is involved in PGCC formation and regulates PGCC senescence, resulting in resistance to Doc (Zhao et al., 2023). Thus, targeting IL-1β in PGCCs could present a promising strategy to combat Doc chemotherapy resistance.

Studies in HCT116 and MCF7 cells suggest that increased ploidy during cellular senescence may be caused by varying levels of mTOR and/or Pim-1 kinase (Mosieniak et al., 2015). Elements such as Cdk1, ROS, and p21, necessary for PGCC formation or survival, might help evade senescence after chemotherapy (Wang et al., 2013; Mosieniak et al., 2015; Galanos et al., 2016). Research shows that upregulation of the prelamin A double mutant S22A-progerin can trigger senescence in PGCCs while blocking nuclear membrane breakdown can impede it (Moiseeva et al., 2022). Polyploidization in senescent cells hinders growth, but PGCCs can generate aggressive tumor cells by reversing polyploidization (Moiseeva et al., 2022).

### 3.7 PGCCs and distinct biophysical phenotypes

PGCCs exhibit key structural features that influence their physical traits, such as higher levels of actin polymerization and vimentin intermediate filaments (VIFs), increased nuclear and cytoskeletal stiffness, enhanced traction, and greater migratory endurance (Xuan et al., 2018). Further research is needed to explore the unique physical characteristics and movement patterns of PGCCs, closely linked to cancer spread (Bravo-Cordero et al., 2012). Studies suggest that changes in the RhoA-Rock1 signaling pathway and actin cytoskeletal network may alter PGCCs' physical traits (Xuan et al., 2018). These cells display increased cytoplasmic and nuclear rigidity to withstand mechanical and chemical pressures. Although PGCCs have stiffer nuclei, they are also more prone to deformation, making them a highly invasive subgroup due to their increased traction (Xuan et al., 2022).

VIFs are integral to maintaining cellular architecture and ensuring structural stability. Jamney et al. found that disrupting the VIFs gene in mouse embryonic fibroblasts resulted in smaller cell size and reduced nuclear volume, highlighting VIFs’ role in providing mechanical support to protect the nucleus during migration (Patteson et al., 2019). PGCCs leverage VIFs to endure axial strain and orient their multinuclear structures along the migration axis, thereby safeguarding their nuclei from substantial deformations (Xuan et al., 2022). The VIF network also influences nuclear alignment, which is crucial for generating daughter cells via amitotic budding (Terriac et al., 2019).

VIFs also play a crucial role in polarizing cell migration. They coordinate microtubule patterns and orient tensile forces during movement (De Pascalis et al., 2018). VIFs are connected to actin and microtubule networks, providing mechanical support essential for the distinctive physical characteristics of PGCCs. This connection aligns the cell’s cytoplasmic structure, enhances movement orientation, and directs migration (De Pascalis et al., 2018; Xuan et al., 2018). PGCCs exhibit significantly higher levels of cytoplasmic VIFs compared to non-PGCCs, with more dispersed and evenly distributed filaments. During migration, PGCCs show a highly polarized VIF network and rely heavily on it to maintain polarity (Xuan et al., 2020).

## 4 Therapies targeting PGCCs

**FIGURE 2:**
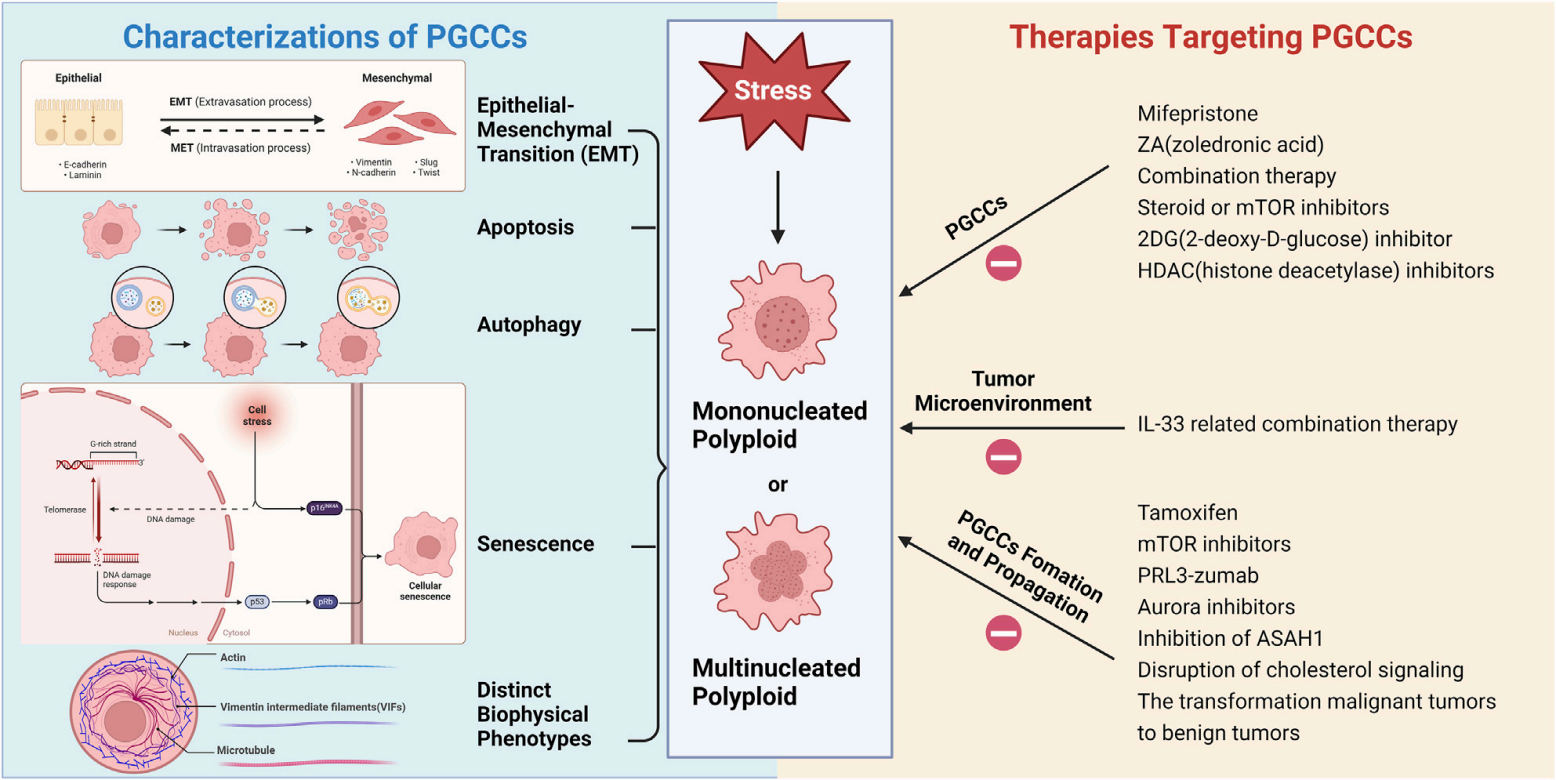
Various characterizations of PGCCs and therapies targeting PGCCs (Created with BioRender.com).

Figure 2 illustrates the various characteristics of PGCCs and therapies targeted at these cells. Increasingly, scientists believe that PGCCs are linked to cancer recurrence and resistance to chemotherapy post-treatment, potentially leading to a poor prognosis for cancer patients (Zhang et al., 2014a; Lopez-Sánchez et al., 2014; Niu et al., 2016). Tagal and Roth discovered that Aurora Kinase (AURK) is a critical factor in the transition from the proliferative cell cycle to polyploid growth in NSCLC and that inhibiting AURK activity can block cell cycle progression (Tagal and Roth, 2021). Consequently, the formation of PGCCs may be a significant cellular response to AURK inhibitors, potentially leading to treatment resistance or tumor recurrence. Recently, H. Xu et al. found that Sirtuin1 (SIRT1), a type III deacetylase, is overexpressed in the cytoplasm and promotes drug resistance in ovarian cancer cells after paclitaxel treatment by increasing PGCC formation and allowing cells to escape senescence (Xu et al., 2023). Additionally, cytoplasmic SIRT1 may play a role in polyploidization by promoting the binding of cyclin B and CDK1 (Xu et al., 2023). Ovarian cancer cells gain a survival advantage through polyploidization, which is a key mechanism for generating chemotherapy resistance. Thus, cytoplasmic SIRT1 represents a novel therapeutic target associated with PGCCs. Future research should explore SIRT1, its binding partners, and related signaling pathways to better understand the mechanisms of resistance induced by SIRT1 overexpression.

PGCCs play a detrimental role in cancer dormancy and reactivation, leading to disease recurrence. PGCCs enter dormancy through aberrant cell cycles mediated by P53 and CDK inhibitors and can exit dormancy by producing daughter cells (Jiao et al., 2024). A recent study by Zheng et al. found that after CoCl_2_ treatment, the expression of Cdc42 and PAK1 increased, promoting the nuclear expression of phosphorylated stathmin (STMN1). This process enhances the proliferation, invasion, and migration of PGCCs and their daughter cells through cytoskeletal remodeling (Zheng et al., 2023). Targeting PGCCs and their progeny can thus be a strategic direction. It has been shown that both vimentin SUMOylation and upregulation of P62 can promote migration and invasion of daughter cells derived from PGCCs by modulating the expression of vimentin nuclear translocation, CDC42, cathepsin B, and cathepsin D (Fan et al., 2023). Targeting P62 and vimentin nuclear translocation may be effective in preventing the generation of PGCCs and their daughter cells. Recent research indicates that interleukin-33 (IL-33) is a key factor in the formation of PGCCs and is crucial in tumor polyploidization. IL-33 can induce polyploidization of tumor cells, leading to tumor progression and metastasis, making it a potential biomarker and drug target for cancer treatment (Kudo-Saito et al., 2020). Additionally, high mobility group box 1 protein (HMGB1), secreted by dying cancer cells through a paracrine mechanism, is involved in radiation-induced PGCCs promoting cancer regeneration via neosis (Zhang Z. et al., 2021). Further exploration of HMGB1’s regulatory mechanisms and the effects of PGCC-derived neosis on cancer metastasis could provide new insights into post-treatment cancer recurrence. Moreover, receptor-interacting protein kinase 1 (RIPK1) has been found to promote the formation of autophagy-dependent dormant PGCCs through activation of the AMPK-mTOR pathway. Inhibiting autophagy prior to chemotherapy could prevent the formation of dormant PGCCs and improve patient prognosis (You et al., 2022). Targeting autophagy can also chemosensitize PGCCs to conventional anticancer drugs. Recent advances in the study of autophagy and senescence in PGCCs suggest that autophagy induction and degradation are necessary to overcome senescence and polyploidy (Patra et al., 2024). Therefore, exploring potent treatments targeting autophagy to block the formation of treatment-induced dormant PGCCs is essential.

Understanding the pathways of PGCC formation may facilitate the development of targeted therapies. Studies have shown that mifepristone can block PGCC formation by binding to the endoreplication protein Chk2, though it may cause side effects (Kapperman et al., 2018). Investigating immune biomarkers associated with PGCC function can advance therapeutic approaches (Senovilla et al., 2012). However, dynamic changes in the tumor microenvironment may limit the effectiveness of immunotherapy in clinical settings (Scarfò and Maus, 2017). Cell cycle checkpoints and DNA repair form a complex network of pathways that maintain cellular homeostasis and regulate genomic integrity (Mirzayans et al., 2013). PGCCs may possess unique phenotypes and vulnerabilities due to their distinct division mechanisms, making cell cycle checkpoints a viable therapeutic target, such as inhibiting PGCCs in the G0 phase. Recently, ST-401, a mild inhibitor of microtubule assembly, has emerged as a promising therapeutic approach to prevent cancer cells from developing into PGCCs. ST-401 increases mitochondrial fission and decreases energy metabolism, inducing interphase death in cancer cells (Vicente et al., 2024). Additionally, PGCCs exhibit significant mitochondrial enlargement and reduced ATP production, likely due to mitochondrial autophagy activation following mitochondrial damage during cancer treatment (You et al., 2022). This finding suggests mitochondria-associated therapeutic strategies against dormant PGCCs in clinical patients.

Meanwhile, the development of treatments for PGCCs can be facilitated by studying metabolic biomarkers related to PGCC function. Drugs such as steroids and mTOR inhibitors show promise in treating PGCCs (Mirzayans et al., 2013). Disruption of the cholesterol signaling pathway is suggested as a promising strategy to block PGCC progeny formation (White-Gilbertson et al., 2022). PGCCs are affected by glycolytic mechanisms controlled by the mTOR pathway. Metformin, resveratrol, and aspirin are proposed as potential anti-PGCC medications that block the mTOR signaling pathway through the activation of AMP-activated protein kinase (AMPK) (Lissa et al., 2014). Additionally, 2-deoxy-D-glucose may reduce PGCCs by inhibiting glycolysis (Donovan et al., 2014). Sphingolipid metabolism also plays a role in PGCCs. Sphingolipids contribute structurally to the plasma membrane and regulate various cell functions (Parveen et al., 2019). During polyploidization and depolyploidization, p21 is crucial upstream of acid ceramidase (ASAH1). Blocking p21 expression with UC2288 during therapy stress can inhibit ASAH1 activation, eliminating PGCCs and their daughter cells, thereby improving therapeutic efficacy (White-Gilbertson et al., 2024). Therapeutic strategies to eliminate PGCCs through p21 include senolytics such as Navitoclax or drugs that disrupt depolyploidization like simvastatin or autophagy regulators (Abbas and Dutta, 2009; Bowers et al., 2022; White-Gilbertson et al., 2022). Sphingolipid metabolism is vital in the mitotic cell cycle of PGCCs, suggesting that targeting this pathway could lead to innovative therapeutic strategies by addressing sphingolipid dysregulation in PGCCs (Lu et al., 2021; Voelkel-Johnson, 2022). Tamoxifen can suppress ASAH1, reducing PGCC progenies in prostate cancer, glioblastoma, and melanoma cells, offering clinical advantages unrelated to estrogen signaling in multiple cancer types (White-Gilbertson et al., 2020). Future clinical trials may consider using tamoxifen as a first-line therapy to inhibit PGCC progeny production. Zoledronic acid (ZA), a drug targeting osteoclasts, inhibits PGCCs by altering their lipid metabolism, reducing PGCCs after cisplatin treatment (Adibi et al., 2023). Cells become resistant to DNA damage and avoid apoptosis during prolonged genotoxic stress when PRL3 is overexpressed, as it inhibits ATM-mediated DNA damage response (Thura et al., 2021). PRL3-zumab specifically targets dormant cancer cells, deactivating chemo-resistant PGCCs with self-renewal capabilities (Thura et al., 2021).

The primary reason for the failure of current cancer treatments is the presence of PGCCs, which exhibit distinct biophysical and metabolic characteristics. This necessitates further exploration of the epigenetic reprogramming mechanisms underlying PGCC dormancy. Zhou et al. proposed a single-cell morphological analysis pipeline for accurate quantification of PGCC populations and identified a selective PGCC inhibitor, Thiostrepton, along with three types of compounds capable of killing PGCCs: ferroptosis inducers, HDAC inhibitors, and proteasome inhibitors (Zhou et al., 2023). This single-cell morphological study approach enables the exploration of effective anti-PGCC treatments across a wide range of malignancies, enhancing the development of the PGCC field. Despite the potential of combining anti-PGCC therapy with existing anti-cancer drugs, further investigation is needed to eliminate the possibility of PGCC relapse after treatment.

## 5 Conclusion

Previous studies suggested that PGCCs are senescent and non-proliferative, but recent research has revealed their unusual properties and functions. Various stresses, such as chemotherapeutic agents, radiation, hypoxia, and pollution, can induce PGCC production. PGCCs can exhibit CSC markers, display the EMT phenotype, and facilitate cancer cell invasion and metastasis. They can also acquire therapeutic resistance and lead to tumor recurrence. In tumor research, PGCCs represent both a challenge and an opportunity. They are markers of tumor progression and drug resistance, yet their early embryonic-like life cycle offers potential therapeutic targets. Focusing on the metabolic pathways and endoreplication of PGCCs in drug development could be crucial to reducing drug-resistant offspring. Identifying specific target molecules involved in polyploidization/de-polyploidization and elucidating their mechanisms of action are also essential. Additionally, oncogenic viruses can induce polyploidy, and targeting these viruses to block PGCC formation may have significant clinical potential (Mirzayans et al., 2018).

Although progress has been made in understanding the role of PGCCs in tumor development, invasion, and metastasis, further research is needed on the molecular mechanisms underlying PGCC formation and the identification of reliable molecular markers for screening and therapy. Future efforts should focus on investigating the unique growth patterns of PGCCs and their biomarkers to identify potential molecular targets for clinical use, aiding in the discovery of novel diagnostic and treatment options. Additionally, exploring whether polyploid cells resulting from cell fusion experience distinct pressures and mechanisms controlling cell demise, as well as analyzing cancer specimens to determine the impact of cannibalism in polyploid tumors, is essential. Understanding erythropoiesis from PGCCs will deepen insights into the tumor vasculogenic mimicry (VM) process and tumor-derived erythropoiesis pathways, leading to new therapies related to tumor VM. Research on PGCCs and senescence can be directed towards reducing senescence-related carcinogenic transformation. Further studies are also needed to explore the mechanisms linking apoptosis and cellular senescence in PGCCs. By investigating the molecular mechanisms leading to the dormancy and reawakening of PGCCs, better insights into cancer recurrence and drug resistance can be gained. Addressing recurrence and metastasis after cancer treatment requires research on clinical interventions targeting PGCCs. Increasingly, research will examine the molecular formation of PGCCs, uncovering the intricate processes involved in their role in tumor formation and growth.
